# Cognitive-motor interference in multiple sclerosis revisited: a dual-task paradigm using wearable inertial sensors and the Paced Auditory Serial Addition Test

**DOI:** 10.3389/fneur.2025.1546183

**Published:** 2025-03-17

**Authors:** Lea Kremer, Lucas Schreff, Daniel Hamacher, Patrick Oschmann, Veit Rothhammer, Philipp M. Keune, Roy Müller

**Affiliations:** ^1^Departments of Neurology and Orthopedic Surgery, Klinikum Bayreuth GmbH, Bayreuth, Germany; ^2^Universitätsklinikum Erlangen, Friedrich-Alexander-Universität Erlangen-Nürnberg (FAU), Erlangen, Germany; ^3^Department of Sports Science, Friedrich Schiller University Jena, Jena, Germany; ^4^Department of Cognition, Emotion and Neuropsychology, Otto-Friedrich-University, Bamberg, Germany; ^5^Bayreuth Center of Sport Science, University of Bayreuth, Bayreuth, Germany

**Keywords:** multiple sclerosis, MS, 25-foot-walk, inertial sensors, cognition, executive functions, Paced Auditory Serial Addition Test (PASAT)

## Abstract

**Introduction:**

Multiple sclerosis (MS) is a chronic autoimmune disease affecting the central nervous system, leading to motor and cognitive impairment. These impairments become especially evident during dual-tasks, such as walking while performing a cognitive activity. Previous research has highlighted changes in gait-specific parameters during dual-tasks, but the cognitive component remains underexamined in MS. This study aims to expand on prior findings by using wearable inertial sensors and the Paced Auditory Serial Addition Test (PASAT) to evaluate the effects of dual-tasks on gait and cognitive performance in persons with MS (PwMS) compared to healthy controls.

**Methods:**

Eighty-six adults (54 PwMS and 32 healthy controls) participated. PwMS were further divided into groups with lower (MS_LCP) and higher (MS_HCP) cognitive performance based on performance on the Symbol-Digit-Modalities Test (SDMT). Gait parameters were assessed using wearable inertial sensors during single- and dual-task 3-min-walking. Statistical analyses compared gait and cognitive performance across conditions and groups.

**Results:**

Under dual-task conditions, PwMS showed significant changes in all gait parameters, including reduced walking speed, stride length, percentage of swing phase and toe clearance, and increased stride time and percentage of stance phase compared to single-task condition. However, under dual-task condition in PwMS only walking speed, stride length and stride time differed from healthy controls. MS_LCP exhibited greater changes in both gait and PASAT performance than MS_HCP and healthy controls. While MS_HCP showed gait parameters comparable to healthy controls during single-tasks, deficits became apparent during dual-tasks. Correlations revealed strong associations between SDMT and PASAT scores but weak links between cognitive and self-reported measures.

**Discussion:**

The findings confirm that dual-task conditions exacerbate gait impairments in PwMS, particularly in those with lower cognitive performance. The use of PASAT as a dual-task cognitive challenge was feasible and had a considerable influence on gait. Results support the capacity sharing theory, suggesting that limited cognitive resources are redistributed between tasks under dual-task conditions.

## 1 Introduction

Multiple sclerosis (MS) is an autoimmune, inflammatory disease of the central nervous system that results in the demyelination of nerves (1–3). The disease affects approximately 2.8 million individuals globally (4), manifesting in a wide range of symptoms. These symptoms include motor (5–8) and cognitive (9, 10) impairments, which are particularly prevalent in everyday activities, for example walking while formulating a text message or searching for specific food items during walking through the grocery store.

To better understand the effects of these impairments, numerous studies have been conducted in which persons with MS (PwMS) are given a motor task [e.g., timed up and go test, 25-foot-walk, 6-min-walk (11–14) or walk over different surfaces (15)], or a cognitive task [e.g., serial seven, digit span or reciting every second letter of the alphabet (15–18)], or both a combination of a motor and cognitive task, known as dual-tasking (17). Recent research of PwMS has shown that performing a cognitive task while walking has an effect on gait-specific parameters. In particular, reductions in walking speed and stride length have been observed during dual-task walking (19–21). However, when it comes to other gait parameters, such as stance phase or toe clearance, results of the concurrent literature are mixed. Some studies describe a greater percentage of stance phase compared to swing phase during dual-task conditions (22–24), whereas no differences were observed in others.

In a systematic review of 20 dual-task studies, Wajda and Sosnoff (21) concluded that the motor component was predominantly evaluated, while the cognitive component was either not considered at all or only evaluated as a secondary aspect. This imbalance in research is particularly problematic as the cognitive symptoms lead to significant limitations in professional, social and personal domains, as well as negatively impacting quality of life and disease progression. Furthermore, due to the lack of treatment options for this symptom group (25–27), it is important to research the pathophysiology and further therapeutic options. Although the systematic review of Wajda and Sosnoff (21) was published in 2015, there have only been few new studies that have examined a potentially detrimental effect on cognition during dual-task paradigms in more detail since then (28–32). In one of these studies, Hsu et al. (33) used a cognitive measure based on the symbol-digit-modalities test (SDMT), to divide the participants into two groups, one exhibiting lower and one exhibiting more elevated levels of cognitive impairment. The motor component was assessed using the 25-foot walk. The dual-task was to perform serial seven subtraction while walking on an electronic walkway. The results demonstrated that the group with lower cognitive performance had significantly higher dual-task costs, mainly in temporal rather than spatial gait parameters.

To address the predominant focus on motor tasks in dual-task MS research, we designed a study to assess the cognitive aspects of dual-task performance. Similar to Hsu et al. (33), participants were divided into two groups based on their cognitive performance as assessed by the SDMT. One group consisted of individuals with higher cognitive baseline performance, while the other group consisted of individuals with lower cognitive baseline performance. In addition, control tests were performed with healthy participants. The motor task was a 3-min-walk during which portable inertial sensors were applied, which are highly sensitive for detecting gait errors (14, 34–36). The cognitive task was the Paced Auditory Serial Addition Test (PASAT), which assesses auditory information processing and calculation ability.

## 2 Materials and methods

### 2.1 Participants

A total of 86 adults between the ages of 18 and 65 years were recruited in the Department of Neurology, Klinikum Bayreuth GmbH, Medical Campus Upper Franconia, Germany (Table 1). Of these, 54 were persons with a verified MS diagnosis (PwMS) according to McDonald criteria (37). The remaining 32 were healthy control subjects. It was necessary for participants to be able to walk twice for 3 min without the use of a walking aid. Further exclusion criteria were hearing impairment, severe cognitive and motor disorders. Besides PwMS were not included in case of a relapse in the last 4 weeks or an Expanded Disability Status Scale (EDSS) >5. All participants provided their written informed consent after they were fully informed about the research protocol, which was approved by the ethical review board of the Otto-Friedrich-University Bamberg, Germany (2023–02/11) and was in accordance with the Declaration of Helsinki.

**Table 1:** Demographic and clinical characteristics of the sample.

		PwMS	Healthy controls
Sex [f/m]	PwMS	34/20	20/12
	MS_LCP	15/7	
	MS_HCP	19/13	
Age [years]	PwMS	42.41 ± 12.27	41.09 ± 14.15
	MS_LCP	42.59 ± 13.51	
	MS_HCP	42.28 ± 11.84	
Height [cm]	PwMS	171.09 ± 9.55	171.44 ± 7.74
	MS_LCP	168.64 ± 10.28	
	MS_HCP	172.78 ± 8.78	
Weight [kg]	PwMS	78.73 ± 22.21*	67.72 ± 13.38
	MS_LCP	76.34 ± 22.86	
	MS_HCP	80.38 ± 21.98*	
EDSS	PwMS	2.08 ± 1.37	n.a.
	MS_LCP	2.45 ± 1.20	
	MS_HCP	1.81 ± 1.44	
MS subtype	PwMS	RRMS = 49, PPMS = 2, SPMS = 3	n.a.
	MS_LCP	RRMS = 20, PPMS = 1, SPMS = 1	
	MS_HCP	RRMS = 29, PPMS = 1, SPMS = 2	

Values of age, height, weight and Expanded Disability Status Scale (EDSS) are expressed as mean ± standard deviation. Significant differences of PwMS (*N* = 54), subgroup MS_LCP (*N* = 22) and subgroup MS_HCP (*N* = 32) from healthy controls (*N* = 32) is indicated with ‘*’ (*p* < 0.05). MS subgroups were established using a split based on SDMT scores <25%. MS, Multiple Sclerosis; PwMS, Persons with MS; RRMS, Relapsing–remitting MS; PPMS, primary progressive MS; SPMS, Secondary progressive MS.

### 2.2 Procedure

The assessment took place in the Gait- and Locomotion Lab of the Klinikum Bayreuth GmbH, Medical Campus Upper Franconia, Germany. Measurements were always taken in the same order. First, a baseline assessment was implemented by means of the SDMT. Subsequently, a gait single-task (motor task) was performed. Afterwards, the PASAT as a cognitive task was administered as a single-task, while patients were sitting down. Finally, both, i.e., the motor task and the cognitive task were completed simultaneously in a dual-task condition.

#### 2.2.1 Motor task (3-min-25-foot-walk)

**Figure 1:**
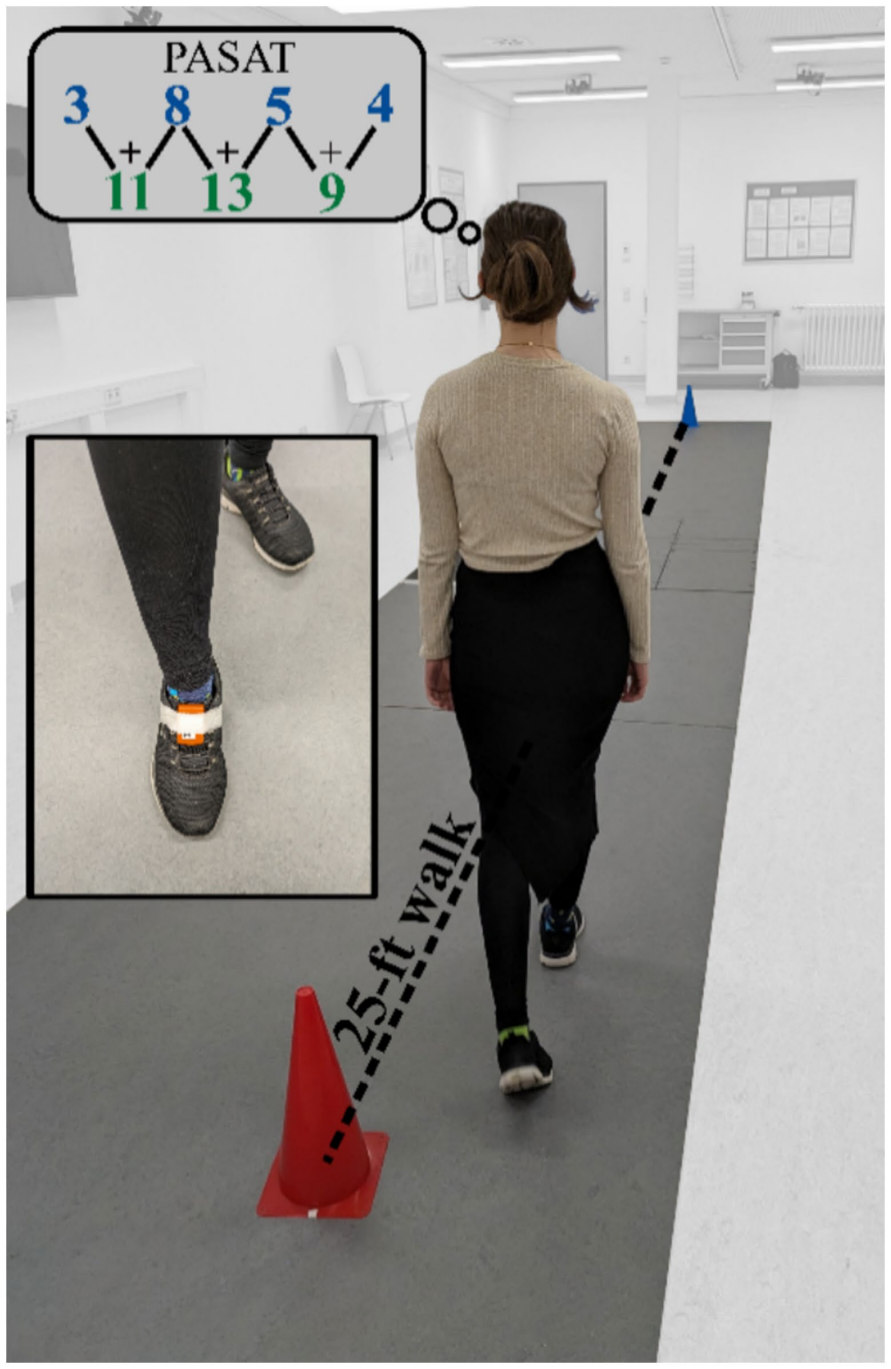
Experimental setup. The flat test track for the motor task is marked by two cones with a distance of 25 feet between them. The wearable inertial sensors are attached to the footwear via the use of an adhesive tape. In the thought bubble, the blue numbers represent the PASAT numbers of the auditory stimulus, and the green numbers represent the participant’s results.

During the motor task, participants were required to repeatedly walk around two cones (25 feet apart) on the flat test track for a period of 3 min. During the 3-min-walking-test, a wearable inertial sensor (MTw2, Xsens technologies B.V.; angular velocity range ± 1,200 deg/s; frequency 100 Hz) was used to assess various gait parameters such as walking speed, stride time, stride length and minimum toe-to-floor distance (MTC). The sensor was attached to the forefoot of the dominant leg [i.e., the foot they would take to kick a ball; (14, 38)] with adhesive tape (Figure 1). To exclude effects of acceleration and deceleration the first and the last 25 feet distances, as well as the first and the last 2.5 m of each distance between the cones were excluded from the following analysis. We used a validated algorithm (39, 40) to calculate mean gait parameters (i.e., walking speed, stride length, stride time, the duration of the stance, the duration of swing phase and MTC).

#### 2.2.2 Cognitive task (PASAT)

The cognitive task was evaluated using the 3 s version of the PASAT (41). This test assesses auditory information processing speed, working memory and executive attention control on a continuous arithmetic task (42). For this purpose, numbers from one to nine were presented auditorily with an inter-stimulus-interval of 3 s. The task was to add the currently presented number to the previous number (Figure 1). As indicated in section 2.2., the PASAT was administered in a neutral condition, while the patient was sitting on a chair, as well as in the dual-task condition, while walking. As previously described, the participants walked around the two cones for 3 min. However, this time, the PASAT must be completed simultaneously.

#### 2.2.3 Symbol-Digit-Modalities-Test (SDMT)

All participants were tested with the oral version of the Symbol-Digit-Modalities-Test [SDMT (43)], prior to the implementation of the single- and dual-task experimental paradigm. The SDMT is used to measure cognitive processing speed and executive attention [e.g., (44)]. For this purpose, participants received a laminated paper which included a legend of digits from one to nine, each paired with a distinct symbol. Additionally, a random sequence out of these symbols was presented. Participants were given 90 s to assign the corresponding numbers to the symbols in the sequence and to verbally name. Furthermore, the SDMT was used to divide PwMS into two subgroups, PwMS with lower cognitive performance (MS_LCP; SDMT <25.0%) and, PwMS with higher cognitive performance (MS_HCP; SDMT≥25.0%) (Table 1). In determining the cut-off value (25.0%), the present study drew upon the findings of a preceding study (45), ensuring that both groups contained a sufficient number of participants for meaningful comparison.

#### 2.2.4 Self-report measures

Self-report measures were used to assess cognitive performance in participants’ everyday lives. These were completed independently by the seated participants in a quiet atmosphere prior to the practical tests. For this purpose, the Scale for the Assessment of Attention Problems by Self- or External Assessment [SEA-R (46)] was utilized. The SEA-R questionnaire contains 33 questions for self-assessment. Each question can be answered on a Likert scale with one of five possible answers (zero to four). Zero corresponds to the answer option “never,” followed by the answer options “rarely,” “sometimes,” “most of the time” and “always.” Furthermore, different components associated with depression were evaluated [CES-D (47, 48)], including emotional, motivational, cognitive, somatic, and interactional factors.

### 2.3 Statistical analysis

Statistical analyses were performed with SPSS 20 (Chicago, IL, USA). To test normality of distributions, Kolmogorov–Smirnov tests were implemented for all gait parameters (i.e., walking speed, stride length, stride time, the duration of the stance, the duration of the swing phase and minimum toe-to-floor distance) as well as the cognitive performance (PASAT) during single- and dual-task. The homogeneity of variance was tested using Levene’s test. Differences between PwMS and healthy controls were assessed by an independent t-test. For the data that were not normally distributed (see Table 2) a Mann–Whitney-U-test was assessed. To evaluate the effect of a cognitive-motor dual-task on the gait parameters and the cognitive performance, we performed a paired t-test for normally distributed parameters or a Wilcoxon test for not normally distributed parameters. To examine the assumed association between cognitive parameters (i.e., SDMT, PASAT) and self-report-measures (i.e., CES-D, SEA-R) Pearson and spearman correlation coefficients were calculated. Participant characteristics were compared using Pearson’s Chi-square for gender and independent t-Tests for age, height, weight and EDSS scores, separately for healthy controls, PwMS and both MS subgroups (MS_HCP, MS_LCP). An alpha level of 0.05 was used for all statistical tests.

**Table 2:** Mean cognitive and gait parameters during single- and dual-task conditions.

	Single-task	Dual-task	ST vs DT	DTC
Cognition (PASAT)				
Control	50.5 ± 9.9	49.0 ± 9.3	0.059	2.35 ± 8.34
PwMS	47.0 ± 12.0	47.5 ± 10.7	0.715	−3.09 ± 18.52
MS_LCP	38.5 ± 13.0*^#^	40.2 ± 12.6*^#^	0.231	−7.26 ± 24.94
MS_HCP	52.9 ± 9.8	52.4 ± 4.9	0.536	−0.23 ± 12.02
Walking speed (m/s)				
Control	1.56 ± 0.18	1.50 ± 0.19	0.002	3.68 ± 6.26
PwMS	1.36 ± 0.30*	1.26 ± 0.30*	0.000	7.56 ± 6.58*
MS_LCP	1.23 ± 0.28*^#^	1.13 ± 0.29*^#^	<0.001	8.21 ± 8.34*
MS_HCP	1.46 ± 0.20*	1.35 ± 0.20*	<0.001	7.11 ± 5.13*
Stride length (m)				
Control	1.55 ± 0.14	1.51 ± 0.14	0.000	2.83 ± 3.24
PwMS	1.42 ± 0.20*	1.35 ± 0.20*	0.000	4.36 ± 4.07
MS_LCP	1.30 ± 0.22 *^#^	1.25 ± 0.23*^#^	<0.001	4.13 ± 5.35
MS_HCP	1.50 ± 0.14	1.43 ± 0.14*	<0.001	4.51 ± 2.97*
Stride time (s)				
Control	1.00 ± 0.07	1.01 ± 0.08	0.101	−1.12 ± 3.73
PwMS	1.05 ± 0.11*	1.10 ± 0.14*	0.000	−3.81 ± 4.72*
MS_LCP	1.08 ± 0.14*	1.14 ± 0.17*	<0.001	−4.97 ± 5.92*
MS_HCP	1.04 ± 0.08	1.07 ± 0.09*	<0.001	−3.02 ± 3.57
Stance phase (%)				
Control	54.25 ± 2.25	54.75 ± 2.66	0.018	−0.92 ± 2.19
PwMS	54.59 ± 2.68	55.12 ± 2.89	0.000	−0.96 ± 1.34
MS_LCP	55.54 ± 2.84^#^	56.14 ± 3.17 ^#^	0.019	−1.07 ± 1.75
MS_HCP	53.94 ± 2.40	54.42 ± 2.49	<0.001	−0.88 ± 1.00
Swing phase (%)				
Control	45.76 ± 2.33	45.30 ± 2.63	0.023	1.00 ± 2.51
PwMS	45.40 ± 2.68	44.83 ± 2.93	0.000	1.27 ± 1.96
MS_LCP	44.41 ± 2.84 ^#^	43.71 ± 3.14* ^#^	0.010	1.60 ± 2.64
MS_HCP	46.08 ± 2.37	45.61 ± 2.55	<0.001	1.05 ± 1.30
MTC (cm)				
Control	2.49 ± 0.66	2.31 ± 0.67	0.086	4.82 ± 27.13
PwMS	2.60 ± 0.90	2.35 ± 0.87	<0.001	10.07 ± 10.98
MS_LCP	2.46 ± 0.96	2.24 ± 0.91	0.002	9.94 ± 13.06
MS_HCP	2.69 ± 0.86	2.43 ± 0.84	<0.001	10.17 ± 9.52

All values are expressed as mean ± standard deviation. Significant differences from healthy controls are indicated with ‘*’ (*p* < 0.05). MTC, minimum toe-to-floor distance. Dual-task-costs (DTC) were calculated with ((DT-ST)/ST) * (−100). Significant differences between MS_LCP and MS_HCP are indicated with ‘#’ (*p* < 0.05). Data that are not normally distributed are underlined.

## 3 Results

### 3.1 Differences between single- and dual-task in PwMS and healthy controls

Regarding gait parameters, healthy controls significantly reduced their walking speed by about 4% and stride length by about 2.5% in the dual-task condition (Table 2). Additionally, the percentage of stance phase increased by about 0.5%, while swing phase percentage decreased significantly by about 0.5%. In PwMS, all measured gait parameters changed significantly between single- and dual-task conditions. More precisely, walking speed decreased by about 7.5%, stride length decreased by about 5%, swing phase percentage decreased by about 0.5%, and MTC decreased by about 10%, while stride time increased by about 5% and stance phase percentage increased by about 0.5% (Table 2). The same trends in these parameters were observed in both subgroups. In sum, in both groups, i.e., MS_LCP and MS_HCP, all gait parameters showed significant changes in the dual-task condition, relative to the single-task condition. In contrast, no dual-task effect was observable in case of cognitive performance in any group (i.e., healthy controls, PwMS, MS_LCP, MS_HCP).

### 3.2 Differences between PwMS and healthy controls

Regarding the results of the cognitive PASAT, only the subgroup MS_LCP differed from the control group, performing significantly worse under both single- and dual-task conditions (Table 2). In the motor task, several significant differences were found comparing the individual groups. Gait parameters between healthy controls and the PwMS differed significantly in walking speed, stride length and stride time. The PwMS walked at a lower speed and stride length, and with increased stride time during single- and dual-task (Table 2). In comparison between healthy controls and MS_LCP, significant differences were found in the same parameters during single-task, with an additional significant deviation in swing phase during dual-task. Except for stride time, all significant parameters were lower in the MS_LCP group than in the healthy controls. Compared to the control group, a significant reduction in walking speed was also noted during single-task in the subgroup MS_HCP. Under dual-task conditions, healthy controls and the MS_HCP showed significant differences in walking speed, stride length, and stride time, with MS_HCP exhibiting slower walking speed and reduced stride length, while stride time was shorter in the control group. When comparing both subgroups, MS_LCP differed in walking speed, stride length, stance phase and swing phase, both in single- and dual-task conditions. Specifically, MS_LCP exhibited slower walking speed, shorter steps, a longer stance phase and a shorter swing phase compared to MS_HCP. Furthermore, the PASAT differed between MS_LCP and MS_HCP under both single- and dual-task conditions.

### 3.3 Association between self-report measures and cognitive measurements

The PASAT as single-task demonstrated a high degree of correlation with the PASAT in dual-task in all groups, reflecting high internal consistency of measurements in the dual-task paradigm (healthy controls: *r* = 0.91, *p* = 0.01; *r*_s_ = 0.85, *p* = 0.01; PwMS: *r* = 0.84, *p* = 0.01; *r*_s_ = 0.74, *p* = 0.01; MS_LCP: *r* = 0.83, *p* = 0.01; *r*_s_ = 0.79, *p* = 0.01; MS_HCP: *r* = 0.57, *p* = 0.01; *r*_s_ = 0.51, *p* = 0.01). Additionally, the SDMT was highly correlated with the PASAT in single-task in the groups PwMS (*r* = 0.62, *p* = 0.01; *r*_s_ = 0.70, *p* = 0.01) and MS_HCP (*r* = 0.51, *p* = 0.01; *r*_s_ = 0.57, *p* = 0.01). However, in the group PwMS (*r* = 0.53, *p* = 0.01; *r*_s_ = 0.57, *p* = 0.01), the SDMT demonstrated a high degree of correlation with the PASAT in dual-task. No high correlations were observed between self-report measures and the cognitive measurements (Table 3).

**Table 3:** Mean values of self-report measures and cognitive measurements.

	SDMT	Depression (CES-D)	Attention problems (SEA-R)
Control	57.24 ± 26.69	7.90 ± 6.71	27.06 ± 11.72
PwMS	44.85 ± 36.70	12.92 ± 9.10 *	36.33 ± 23.11
MS_LCP	9.7 ± 7.66^#^	18.25 ± 10.15* ^#^	41.20 ± 28.84*
MS_HCP	69.02 ± 27.98	9.48 ± 6.46	32.98 ± 17.92

All values are expressed as mean ± standard deviation. Significant differences from healthy controls are indicated with ‘*’ (*p* < 0.05). Significant differences from between MS_LCP and MS_HCP are indicated with ‘#’ (*p* < 0.05). Data without normally distribution are underlined.

## 4 Discussion

Our study shows that cognitive performance under dual-task conditions remains unchanged across all groups compared to single-task conditions (Table 2). Regarding the gait parameters, it is noticeable that PwMS with low cognitive performance (MS_LCP) exhibit gait impairment compared to healthy controls under both single- and dual-task conditions. In contrast, in PwMS with high cognitive performance (MS_HCP), certain gait impairments only emerge under dual-task conditions (i.e., significantly reduced stride length and stride time compared to healthy controls). A possible explanation for the preserved cognitive performance despite deteriorated motor performance in MS_HCP under dual-task conditions could be provided by the capacity sharing theory (49–52).

### 4.1 Capacity sharing theory

The capacity sharing theory has been described in several publications (49–52) and postulates that each individual has a limited cognitive capacity which is distributed among various tasks processed in parallel. The assumption is that more demanding tasks require more resources. Consequently, when cognitive capacity is utilized, less cognitive capacity is available for less demanding tasks that are performed in parallel with more demanding tasks than if these tasks were to be completed alone. This results in poorer performance on the easier task, while performance on a simultaneously performed more demanding task remains the same. MS_HCP showed a similar level of performance in single-task compared to healthy controls [only the walking velocity was significantly reduced in MS_HCP (Table 2)]. However, in dual-task scenarios, the cognitive task, as the more demanding task (see section 4.3), appears to take up a significant amount of resources, limiting the ability to maintain the gait pattern as in single-task. Consequently, it can be concluded that cognitive capacity is already limited in MS_HCP compared to healthy controls, but that this difference can only be recognized under dual-task conditions. To provide a comprehensive overview, it is imperative to also examine the MS_LCP, which already showed significant differences in the cognitive task, walking speed, stride length and stride time under single-task conditions, and these differences have been shown to persist under dual-task conditions. This finding suggests that cognitive capacity is already significantly constrained to the extent that single-tasks are sufficient to detect these deficits. However, the results of the capacity sharing theory suggest that dual-task measures are particularly relevant for PwMS whose limitations are not yet so obvious, serving as an early screening tool and providing an objective insight into the progression of the disease, especially in its early stages. The advantage for clinical practice is that dual-task measures can be easily integrated into everyday clinical practice due to its short test duration and the fact that very few materials are required.

### 4.2 The effect of cognitive impairments on gait parameters

A comparison between the gait parameters of MS_LCP and MS_HCP in our study shows significant differences in gait speed, stride length, stance phase and swing phase in both the single- and dual-task conditions. This is compatible with results of Hsu et al. (33), who also reported reduced walking speed in MS_LCP compared to MS_HCP, but only under dual-task conditions. Moreover, no difference was noted in step length in single- and dual-task. This discrepancy may be explained by the use of different measurement systems for the motor task. In our study, all participants were required to walk for a period of 3 min while wearing inertial sensors, which are highly sensitive in the detection of gait disturbances in PwMS even in early stages of MS (11, 14). In the study of Hsu et al. (33) the authors utilized a Zenos Walkway, which has been demonstrated to be moderately to highly effective in the assessment of gait distributions (53). Moreover, each participant was required to walk four times over the 16-foot walkway. An advantage of our motor task might be that it generates a greater quantity of data due to the longer time span over which it is conducted. Furthermore, this also implies that participants must possess higher motor abilities than those observed in the Hsu et al. (33) study. This is reflected in the notably lower Expanded Disability Status Scale (EDSS) scores observed in our study participants. Moreover, in contrast to the study by Hsu et al. (33), in the current work no significant difference was observed in the EDSS between the MS_LCP and MS_HCP subgroups.

### 4.3 Feasibility of the PASAT

As in the study by Hsu et al. (33), the majority of studies utilized subtraction tasks (16, 18, 33, 54) or word finding tasks (13, 22, 55–57). The current study used the PASAT, a test that assesses both auditory working memory and executive attentional control, and has been used to investigate dual-task effects in wheelchair users (58). Compared to other, relatively less demanding cognitive tasks, the PASAT might bind more cognitive resources and thus, influence the gait parameters more strongly under the dual-task condition. Additionally, the PASAT is subjectively perceived as a challenging test by PwMS, which may lead to frustration, particularly in those with more impaired cognitive abilities (28, 42, 59–61). Accordingly, some studies advise against its continued use in neuropsychological testing (62). This also implies that the PASAT is particularly beneficial for PwMS with mild cognitive impairment (MS_HCP) to produce a higher dual-task effect. However, for PwMS with severe cognitive impairment (MS_LCP), the demanding nature of the test could prove overwhelming, potentially leading to increased frustration and discomfort for patients compared to other, less cognitively demanding tasks under dual-task conditions. Furthermore, in the present study, the motor task and the PASAT were initially performed as a single-task to establish a baseline, followed by the dual-task. In accordance with the aforementioned research status, the PASAT, as a cognitive component, could have been subjectively rated as a more challenging task by our participants at the outset of the dual-task condition in comparison to the motor task. This could have resulted in the participants prioritizing the cognitive task under dual-task conditions, despite no predetermined prioritization of the tasks in the study. This would also explain why the cognitive scores did not change significantly under dual-task condition, but some gait parameters did.

### 4.4 Limitations

Despite the significance of the findings, it is essential to acknowledge the methodological limitations of the study. The main limitation of the study is certainly that the order of the tasks was not randomized, which may have led to learning effects, and fatigue, a common complaint among PwMS, was not considered. It would be interesting, for example, to randomize the sequence or to divide the tasks into intermediate results rather than the total time of 3 min, to avoid potential confounding. A further constraint of the study is the weak correlation between the questionnaires and the cognitive measurements. This could be indicative of the fact that neuropsychiatric comorbidities, such as depression, may not be captured by the dual-task measurement and that neuropsychiatric testing is still necessary to assess them. The weak correlation with the SEA-R, which reveals attention deficits and the resulting problems in everyday life, raises concerns that the questionnaire, as a subjective measurement instrument, does not capture the deficits in comparison to the objective dual-task measurement. Furthermore, medication use was not taken into account in this study, despite certain medications being used to treat these motor and cognitive symptoms. This could also have influenced the results. Further investigation of medication use could provide insight into which medications may be effective for these impairments and should be included in future research given the lack of treatment options. Finally, it should be noted that, despite the size of the sample, it is difficult to fully represent such a variable clinical picture. In addition, the sample size could not fully cover all degrees of disability.

## 5 Conclusion and outlook

The findings confirm that single- and dual-task conditions exacerbate gait impairments in PwMS, particularly those with lower cognitive performance. Furthermore, the PASAT was found to be feasible under dual-task conditions and also caused a change in gait performance in cognitively less impaired PwMS due to the high level of attentional resources that were required. It can therefore be concluded that a more intensive cognitive task increases the dual-task effect and should therefore be used in dual-task condition, especially for less cognitively impaired people. Despite the above-mentioned limitations, the results obtained in the present study have a significant impact on the future direction of therapeutic options. Firstly, the findings suggest that dual-task should be explicitly trained (25, 62), as it has been demonstrated that everyday tasks are generally performed under dual-task conditions. This is an important factor to consider, given that dual-task tasks have been shown to pose other challenges and limitations than single-task tasks. Secondly, the results indicate that a combination of motor and cognitive training is necessary, because as demonstrated in the study, cognitive performance influences motor performance under everyday conditions. In the event of cognitive difficulties, it would be necessary to observe a deterioration in motor performance in dual-task tasks, as indicated by the results of the study. Consequently, this component should also be addressed through training. Conversely, in cases of poorer motor skills, it is anticipated that maintaining brain capacity will contribute to supporting motor performance under dual-task conditions.

## Data Availability

The raw data supporting the conclusions of this article will be made available by the authors, without undue reservation.

## Author’s note

The present work was performed in fulfillment of Lea Kremer of the requirements for obtaining the degree “Dr. Med.”
